# Supplementary material for: Physical Activity Capacity Assessment of Patients with Chronic Disease and the Six-Minute Walk Test: A Cross-Sectional Study
Source: Healthcare (Basel). 2022 Apr 19;10(5):758. doi: 10.3390/healthcare10050758 (PMC9141940; doi:10.3390/healthcare10050758)

Supplementary Files

Figure S1. Flow chart of patients

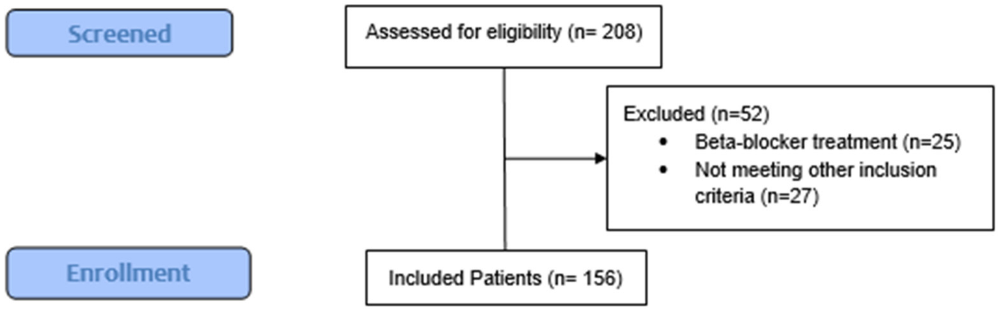

Figure S2. Scatter Graphs exercises capacities as a function of ages and BMI.

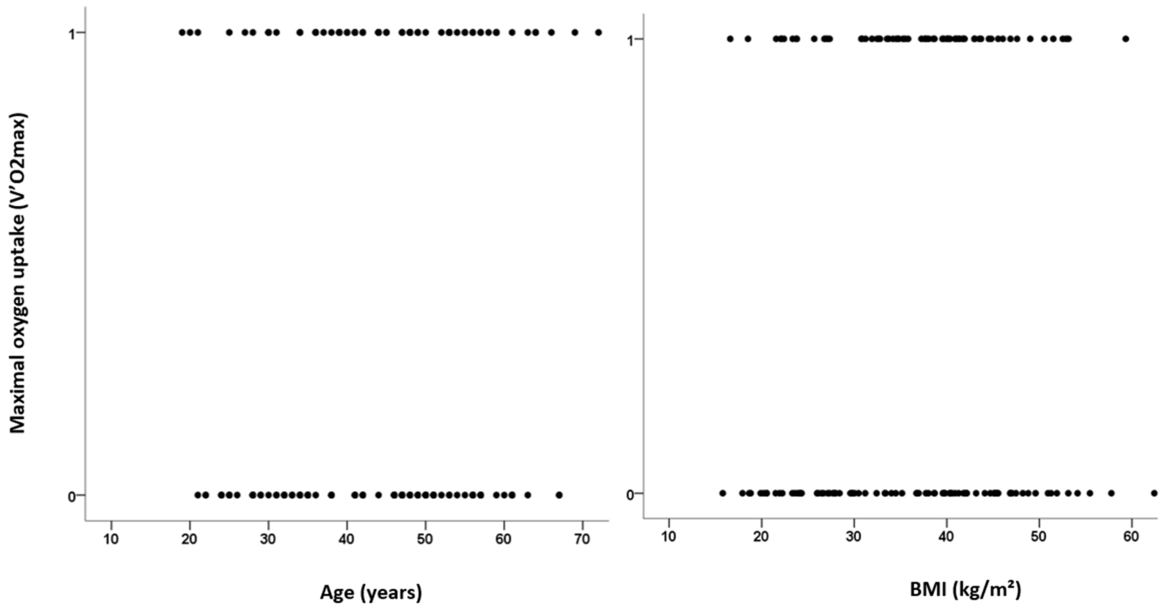

Supplement: Supplementary file 1 [file healthcare-10-00758-s001.zip › healthcare-1657495-supplementary.pdf]
